# Functional genomics screen with pooled shRNA library and gene expression profiling with extracts of *Azadirachta indica* identify potential pathways for therapeutic targets in head and neck squamous cell carcinoma

**DOI:** 10.7717/peerj.6464

**Published:** 2019-03-01

**Authors:** Neeraja M. Krishnan, Hiroto Katoh, Vinayak Palve, Manisha Pareek, Reiko Sato, Shumpei Ishikawa, Binay Panda

**Affiliations:** 1 Ganit Labs, Bio-IT Centre, Institute of Bioinformatics and Applied Biotechnology, Bangalore, India; 2 Ganit Labs Foundation, New Delhi, India; 3 Department of Genomic Pathology, Medical Research Institute, Tokyo Medical and Dental University, Tokyo, Japan; 4 JST, PRESTO, Saitama, Japan

**Keywords:** Drug target, Pooled shRNA screen, Neem, Gene expression, HSF-1, TGF-β, Head and neck squamous cell carcinoma, Azadirachta indica, Anti-tumorogenic, HSC-4

## Abstract

Tumor suppression by the extracts of *Azadirachta indica* (neem) works via anti-proliferation, cell cycle arrest, and apoptosis, demonstrated previously using cancer cell lines and live animal models. However, very little is known about the molecular targets and pathways that neem extracts and their associated compounds act through. Here, we address this using a genome-wide functional pooled shRNA screen on head and neck squamous cell carcinoma cell lines treated with crude neem leaf extracts, known for their anti-tumorigenic activity. We analyzed differences in global clonal sizes of the shRNA-infected cells cultured under no treatment and treatment with neem leaf extract conditions, assayed using next-generation sequencing. We found 225 genes affected the cancer cell growth in the shRNA-infected cells treated with neem extract. Pathway enrichment analyses of whole-genome gene expression data from cells temporally treated with neem extract revealed important roles played by the TGF-β pathway and *HSF-1*-related gene network. Our results indicate that neem extract affects various important molecular signaling pathways in head and neck cancer cells, some of which may be therapeutic targets for this devastating tumor.

## Introduction

Ethnobotanical databases have been widely explored for understanding the historical background, phytochemical and medicinal properties of plants (Schuler, 2004; Abo, Fred-Jaiyesimi & Jaiyesimi, 2008; Sujarwo et al., 2016). However, evidence-based know-how and the mechanism(s) of action of many plant-derived compounds are currently lacking. *Azadirachta indica*, neem, is one such species that is unique, versatile and important. The neem tree is also one of the most intensively studied sources of natural products with plant-derived extracts showing anti-bacterial, anti-fungal, anti-viral, anti-inflammatory, anti-hyperglycaemic, immunomodulatory, anti-malarial, and anti-carcinogenic properties (reviewed in Subapriya & Nagini, 2005). Past efforts on research on neem have mostly been focused on pre-clinical studies, concentrating mainly on two purified neem-derived compounds, nimbolide and azadirachtin, due to their pharmaceutically- and agriculturally-important properties, respectively. The emergence of genome-wide tools and techniques has opened up new avenues to pursue research through systematic studies on cancer drug target discovery using neem-derived metabolites. One such method is RNA interference. It is a powerful and proven system to perturb gene function in higher organisms by phenotypic screening, with the potential ability to identify pathways and networks of genes involved in cancer (Tang et al., 2008; Blakely, Ketela & Moffat, 2011; Poell et al., 2011; Iorns et al., 2012; Mendes-Pereira et al., 2012; Poell et al., 2012; Singleton et al., 2013). Use of pooled genome-wide libraries of short hairpin RNA (shRNA) coupled with next-generation sequencing offers higher sensitivity and a broader, dynamic range to screen for biological activity with the potential to identify pathways for drug actions through network-based approaches (Erler & Linding, 2010; Erler & Linding, 2012).

Head and neck squamous cell carcinoma (HNSCC) is the sixth leading cause of cancer worldwide (Jemal et al., 2011) with a 5-year survival of less than 50% (Mishra & Meherotra, 2014). Patients with locally advanced HNSCC undergo surgery with definitive radiation therapy, with concurrent chemoradiation, and induction therapy used as alternative options where surgery can not be performed. In the recent decade, targeted therapy options have been used for patients with HNSCC (reviewed in Kozakiewicz & Grzybowska-Szatkowska, 2018). The first targeted *in vivo* killing of HNSCC cancer cells was achieved using the epidermal growth factor and cisplatin (Bhirde et al., 2009). Inhibitors targeting epidermal growth factor receptor (EGFR)-regulated pathways, Vascular endothelial growth factor (VEGF) pathway, multiple kinases, PI3-K/AKT/mTOR pathway, and other novel targets, in a combined modality treatment with immunotherapy, are likely to increase therapeutic efficacy in patients with HNSCC (Matta & Ralhan, 2009; Kaidar-Person, Gil & Billan, 2018). Except for oropharyngeal tumors, human papillomavirus (HPV) is rare in oral cavity tumors (Palve et al., 2018). Its role in risk stratification is still under clinical trial evaluations in non-oropharyngeal tumors. Along with viral status, the molecular complexity and tumor heterogeneity have not been factored into HNSCC management (Malone & Siu, 2018). In the past, the ability of neem-derived compounds to stop cell proliferation and their mechanism(s) of action have been reported, including in HNSCC cell lines (Roy et al., 2007; Harish Kumar et al., 2009; Harish Kumar et al., 2010; Priyadarsini et al., 2010; Gupta et al., 2013; Hsieh et al., 2015; Kasala, Bodduluru & Barua, 2015; Patel et al., 2016; Chien et al., 2017; Kowshik et al., 2017). Neem extracts exhibit selective cytotoxicity towards cancer cells compared to normal cells, thereby reducing toxicity during cancer therapy (Di Ilio et al., 2006; Ricci, Berardi & Risuleo, 2008; Kikuchi et al., 2011). In addition, neem compounds such as nimbolide exhibits lower resistance and high efficacy by inhibiting RECKlessness and up-regulating tumor suppressors (RECK group of proteins) that are normally down-regulated during cancer (Kowshik et al., 2017). Neem leaf glycoprotein (NLG) restores the dysregulation of the chemokine signaling in HNSCC cells, and may be thus utilized to design new immunotherapeutic protocol (Chakraborty et al., 2008). However, efforts towards finding novel drug targets and the pathways using genome-wide tools for neem-derived compounds is currently lacking. The current study combined genome-wide shRNA based functional screening and whole-genome gene expression assay using a head and neck cancer cell line, paving ways to characterize and understand the critical molecular pathways for the therapeutic properties of neem leaf extract against HNSCC.

## Materials and Methods

### Neem plant extracts

We tested leaf, bark, fruit, dry seed and twigs from neem plants to make ethanolic extracts. First, the plant parts were collected fresh and thoroughly washed with MilliQ water followed by 70% ethanol and the process was repeated twice with a final wash with MilliQ water before they were air dried under controlled condition for several days. Once the plant organs were dry, 250 mg of each organ was measured and ground separately using a grinder, mixed with 500 ml of ethanol and transferred to a glass bottle. The mixture in the glass bottle was rocked overnight to mix thoroughly and then centrifuged at 5,000 g for 10 min. The supernatant was separated, and the residue at the bottom was again treated with 500 ml of ethanol with overnight rocking at room temperature followed by centrifugation. The process was repeated three times, and the supernatant was pooled from all the three spins before proceeding to the next step. The ethanolic extract (referred as neem extract) was evaporated using an oven at 55 °C till the materials became very viscous and gooey like and then kept at 4 °C until further use.

### Reconstitution of neem extracts

Neem extracts were dissolved in DMSO to make a stock solution, which was serially diluted further with Dulbecco’s Modified Eagles’ Media (DMEM) to make a final concentration of 2 mg/ml as working stock solution. The working stock solution was passed through a 0.22 µm sterile filter and stored until further use. The filtered and sterile stock solution was further diluted with DMEM to make the final solution and was used for treatment.

### Cell culture and IC_50_ calculation

Multiple human HNSCC cell lines (UPCI:SCC029B and UPCI:SCC040, both gifts from Dr. Susan Gollin, University of Pittsburgh, PA, USA (White et al., 2007); UM-SCC47, a gift from Dr. Thomas Carey, University of Michigan, MI, USA (Brenner et al., 2010); HSC-3 and HSC-4, purchased from RIKEN, Japan (Momose et al., 1989) were tested with all neem extracts before HSC-4 was chosen for further study as the cells showed excellent growth inhibitory pattern in the presence of neem extracts. All the cells were maintained in DMEM supplemented with 10% FBS, 1X MEM non-essential amino acids solution and 1X penicillin/streptomycin mixture (Gibco, Billings, MT, USA) at 37 °C with 5% CO_2_ incubation.

For IC_50_ calculation, the xCELLigence Real-Time Cell Analysis (RTCA) DP instrument (Acea Bio, San Diego, CA, USA) was used. The device monitors and quantifies cell proliferation, morphology change, and provides an opportunity to observe the quality of cell attachment real-time. HSC-4 cells (1.0 × 10^4^) were seeded onto wells with varying concentrations of neem extract (25–800 µg/ml) for varying times (0–48 h). The cells were kept under observation, and IC_50_ values were calculated for all extracts and those with DMSO only used as vehicle control. Neem leaf extract showed the best dose response (Fig. S1) and therefore chosen for further study. All subsequent experiments were carried out with the IC_50_ concentration of neem leaf extract unless otherwise specified.

### Whole genome gene expression assays and data analyses

HSC-4 cells were seeded and treated with neem leaf extract (200 µg/ml) at nine pre-defined time points (5 min, 10 min, 15 min, 30 min, 1 h, 3 h, 6 h, 10 h, and 24 h) and rescued with fresh complete medium post 48-hour treatment at these time-points. The samples were assayed for gene expression using Illumina whole-genome HumanHT-12 v4 expression BeadChip (Illumina, San Diego, CA), following the manufacturer’s specifications. RNA quality was checked using Agilent Bioanalyzer with RNA Nano6000 chip for integrity before being used in the gene expression assays. The RNA samples were labeled using Illumina TotalPrep RNA Amplification kit (Ambion, Foster City, CA, USA) and processed following the manufacturer’s recommendations. Gene expression data was collected using Illumina’s HiScan and analyzed with the GenomeStudio (v2011.1 Gene Expression module 1.9.0), and all assay controls were checked to ensure the quality of the assay and chip scanning. Raw gene-wise expression signal intensities from GenomeStudio were transformed, normalized using VST (Variance Stabilizing Transformation) and LOESS methods, respectively, using the R package lumi (Du, Kibbe & Lin, 2008) and further batch-corrected using ComBat (Johnson, Li & Rabinovic, 2007). The pre-processed intensities were subjected to differential expression analyses using the R package, limma (Ritchie et al., 2015) and fold change values were computed for various time-points in the treatment and rescue conditions versus the control samples. Genes that were serially up-regulated or serially down-regulated across at least four consecutive time-points were short-listed for further analysis.

### Genome-wide shRNA lentivirus library screening and transduction

**Figure 1:**
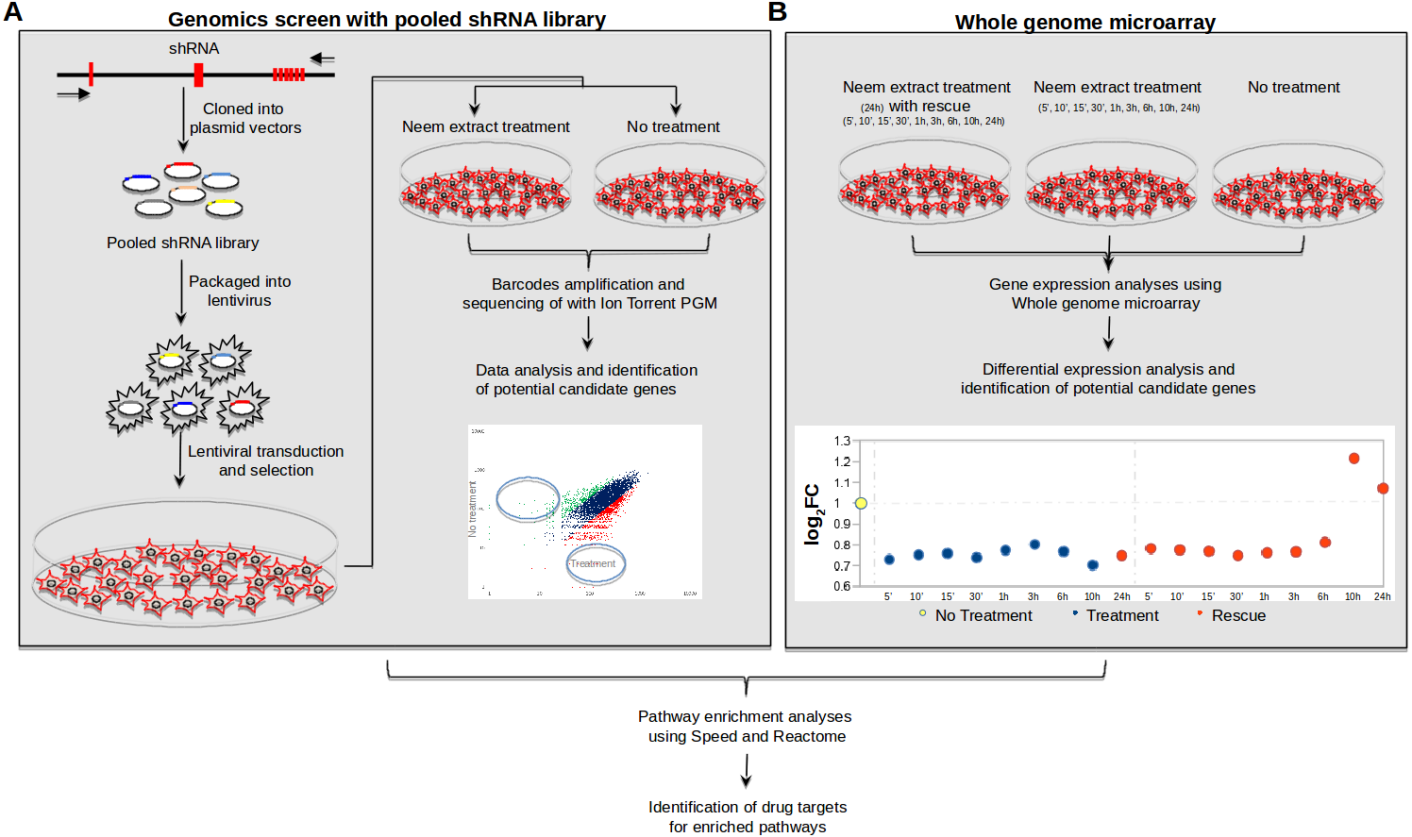
Overview of the screening method. (A) Whole-genome functional shRNA screening experiment, (B) Whole-genome gene expression microarray experiment.

We used a genome-wide shRNA library for signaling pathway genes consisting of 27,500 shRNAs (Human Module 1, Cat No DHPAC-M1-P from Cellecta) for our experiment (Fig. 1). Plating of the cells, transfection, DNAse treatment, viral titer estimation, lentiviral transduction with polybrene and final puromycin selection were carried out following the manufacturer’s instructions. HSC-4 cells were infected with the shRNA lentivirus library and cultured with or without neem leaf extract treatment and harvested, and frozen, followed by the extraction of their genomic DNA (Fig. 1). Two concentrations of neem leaf extract (200 µg/ml and 300 µg/ml) were used for the treatment of cells. The RFP signals at the time of harvesting (72 h after the lentivirus infection and before the puromycin treatment) were confirmed by observing the cells under a fluorescent microscope (Fig. S2).

### Next-generation sequencing post-genome-wide shRNA screening

**Figure 2:**
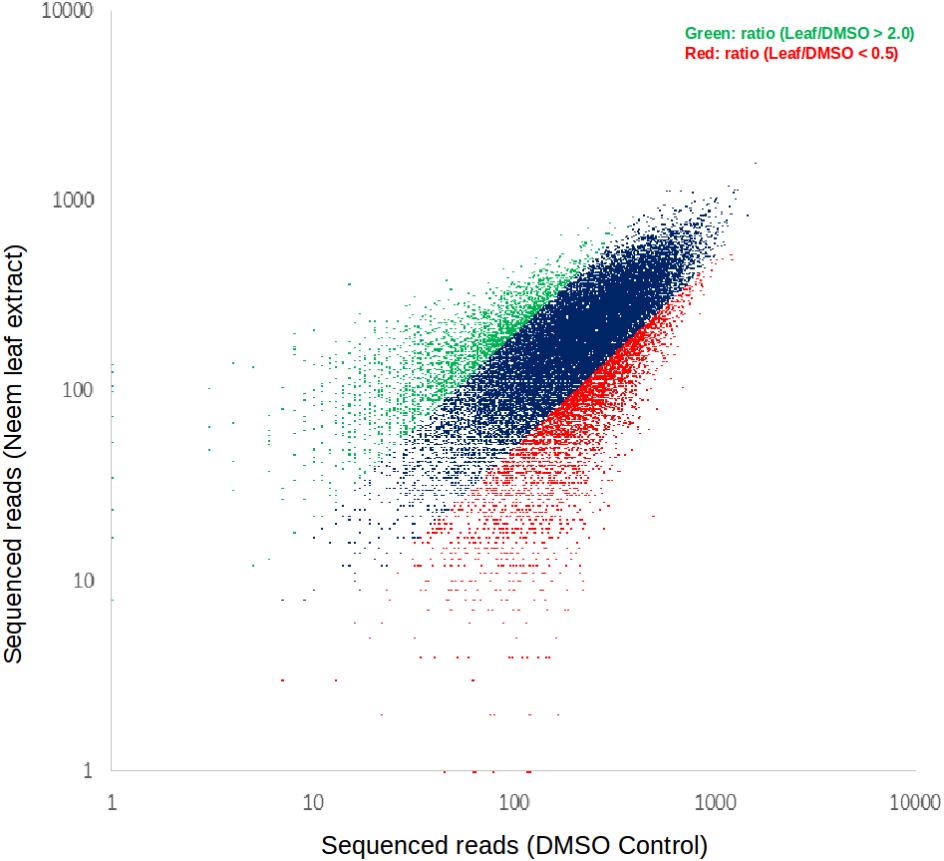
Scatter plot for barcodes in the sequencing reads in treated vs. control neem leaf extract.

We performed PCR amplification of barcode regions of the lentivirus library, followed by sequencing using the PGM system (Ion Torrent; Thermo Fisher, Waltham, MA, USA). Read QC was performed on the raw barcode-assigned reads to filter out reads that contained non-existing shRNA sequences, or were too short (sequencing errors) from both control and shRNA library treated samples. The shRNAs with increased (>2-fold) clone size in neem leaf extract-treated cells compared to the control cells (with DMSO vehicle control) implied that the corresponding shRNAs contributed to the favorable cell survival or growth when treated with neem leaf extract (Fig. 2). On the other hand, the shRNAs whose clone sizes were decreased (<2-fold) in neem leaf extract-treated cells compared to the control cells implied that the knockdown of the corresponding genes worked in synergy with neem leaf extract for unfavorable cell survival or growth (Fig. 2). Genes represented by at least two shRNAs were considered for further analyses to secure the reproducibility of the phenotypes of shRNA knockdown.

### Interaction network, drug targets and pathway enrichment analyses

The short-listed genes from the whole genome expression assay and the consensus list of genes derived from the neem-treated shRNA transfected cells were combined after removal of redundancy, if any. These genes were subjected to pathway enrichment analyses using two independent resources: namely, Speed (Parikh et al., 2010) and Reactome (Croft et al., 2013). Speed enables discovery of upstream over-represented signaling pathways responsible for observed differential expression changes in the genes. Reactome allows merging of pathway identification and over-representation, while allowing interactors to increase the analysis background. We ran Speed while exercising the ‘Signature genes must be unique’ option to identify the significantly enriched pathway(s) first, before running it without choosing this option. This provided us with a complete list of genes perturbed in the enriched pathway(s). The perturbed list of genes from Speed, and the over-represented genes and their interactors from enriched pathways detected by Reactome were subjected to network analyses using cBioPortal (Gao et al., 2013) (http://www.cbioportal.org). Also included with these genes, were 50 significant most affected genomic profiles (mutations, putative copy number alterations from GISTIC and differentially expressed mRNAs (more than 2-fold) in the TCGA HNSCC dataset (provisional dataset)). Reactome was run by exercising the ‘Include Interactors’ option to increase the background list of genes. Cancer drugs were mapped to this network while differentiating between the FDA approved ones from those in the pipeline.

## Results

### IC_50_ calculation of the neem extracts on HNSCC cell line HSC-4

The IC_50_ values for the leaf, bark, fruit, seed and twig extracts were 0.184, 0.226, 2.932, 0.530, and 0.283 mg/ml, respectively, where the leaf extract showed the best dose response (Fig. S1). Therefore, the leaf extract was used in subsequent experiments at IC_50_ concentrations unless otherwise specified.

### Gene expression changes in response to neem leaf extract

The overall approach using a combination of functional genomics screening assay with the genome-wide shRNA library and whole genome expression assay with treatment and rescue conditions is depicted in Fig. 1. To precisely clarify the mode of molecular actions of the neem leaf extract in a time-dependent manner, we investigated the changes in mRNA expression levels of all genes during the neem treatment compared to the steady state (24 h time-course), as well as their behaviors during the following rescue periods (24 h time-course).

**Figure 3:**
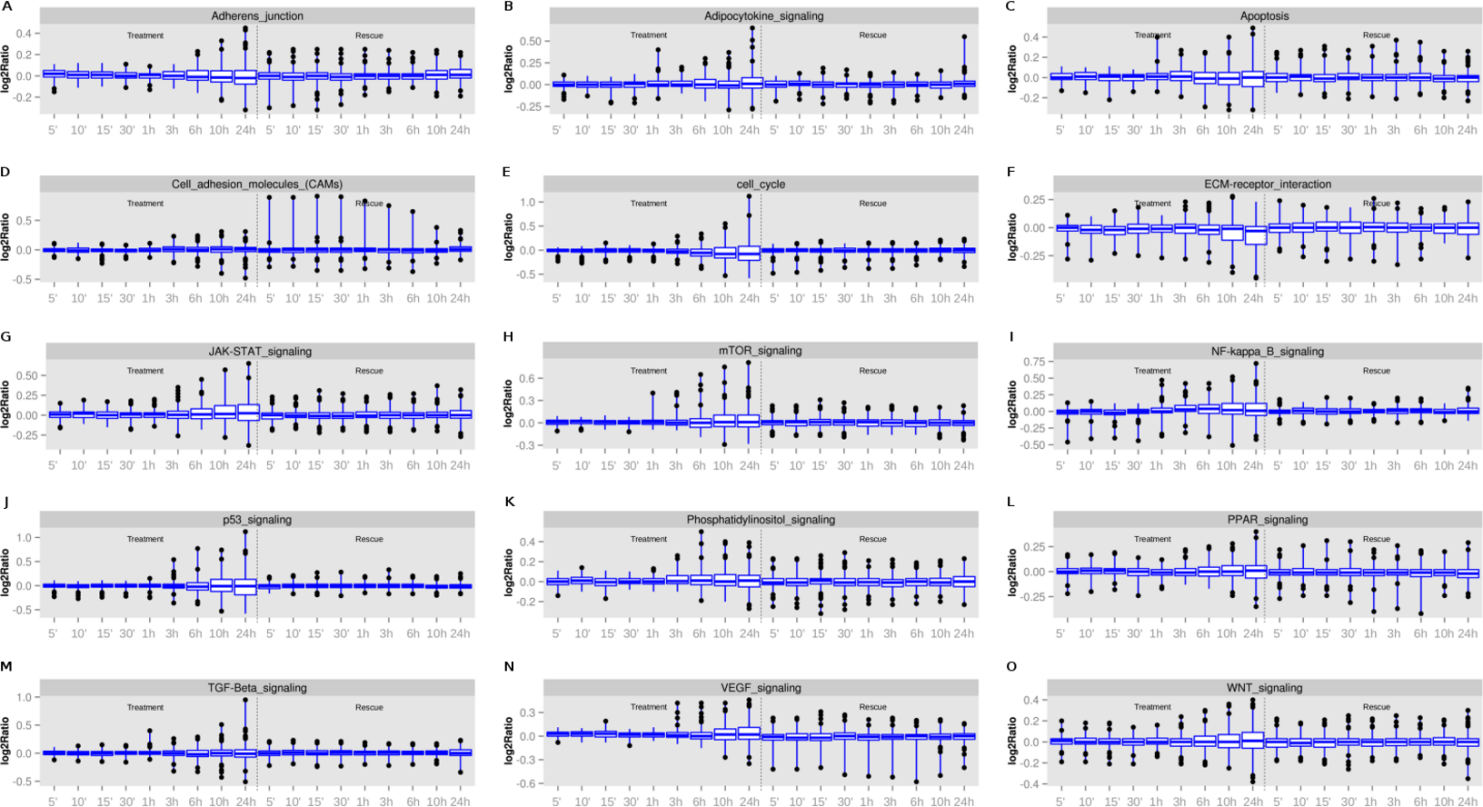
Cancer-related genes and pathways (A–O) affected by neem extract at various time points and with the follow up rescue.

Expression values for all genes at various time points (5 min, 10 min, 15 min, 30 min, 1 h, 3 h, 6 h, 10 h, and 24 h) and the follow-up rescue experiment (for the same periods of time) along with the fold change statistics and pathways affected is provided in Table S1. Expression of 28 genes was altered upon treatment with neem leaf extract, and could not be rescued after withdrawal of the neem leaf treatment (as indicated by a ≤ 0.01 standard deviation across the last treatment time-point and all rescue time-points). Out of those, 8 genes were temporally up-regulated (*CYP46A1*, *PDGFD*, *HOXA3*, *GRIK1*, *TRIM10*, *DDA1*, *OAZ1* and *NAT8B*) (*R* ≥ 0.6), and 20 temporally down-regulated (*KLK12*, *PQLC1*, *LEF1*, *IL15RA*, *CHAC2*, *LMNA*, *EEF1A1*, *EHD4*, *CS*, *COG5*, *MS4A6A*, *FH*, *PRPS1*, *GR14*, *HNRNPM*, *OPRM1*, *KCNIP4*, *KCNC4*, *IMPACT* and *MRAP*) (*R* ≤  − 0.6). We selected genes (*n* = 98:93 up-regulated *and* 5 down-regulated) with altered expression (ratio at least below 0.8 or at least above 1.2) over at least four consecutive time-points (Table S2) for pathway analysis comprising 15 cancer-related pathways (Table S1) for both treatment and rescue conditions (Fig. 3). The usual threshold of at least 2-fold change in expression was relaxed to 1.2-fold, based on the observed fold-change distribution of the results and also that we were selecting differentially expressed genes based on such fold-change differences across four consecutive time-points (Table S2). Genes involved in JAK-STAT, mTOR, VEGF, NF-κ β, and WNT signaling pathways, displayed a cumulative up-regulation effect over time. However, the action of neem leaf extract was reversed upon rescue. Their maximum values for expression change (log_2_ ratios) were 0.75, 0.9, 0.6, 0.75 and 0.4, respectively. Genes involved in the ECM-receptor interaction, on the other hand, were cumulatively downregulated. This action was also completely reversed upon the rescue (Fig. 3). Genes involved in the other nine pathways either showed mixed effects of up- and down-regulation, or marginal differential regulation (Fig. 3).

### Functional genomics with pooled shRNA library with neem leaf extract treatment

A representative result of the shRNA screening is shown in Fig. 2. Sequencing read statistics, and unique shRNA reads obtained after next-generation sequencing post neem leaf extract treatment are provided in Table 1. The results from the PCR amplification of the barcodes, in control and treated cells, showed 1,369 and 1,011 shRNAs with high (ratio versus control: >2) and with low (ratio versus control: <0.5) clonal sizes respectively (Fig. 2). This represented 149 and 78 genes, respectively, where more than one shRNA were involved (Table S4). The direction of changes in clonal size for genes are described in Table S4. To globally obtain a set of genes whose function and/or expression were somehow related to the neem leaf extract treatment, we investigated the gene lists from the two independent screening experiments.

**Table 1:** Sequencing read statistics in functional pooled shRNA screening experiments.

	Total number of barcode-assigned reads	QC-pass reads	%	Number of unique shRNA hits (total 27,495 shRNAs)	%
DMSO control for neem leaf extract	7,658,211	6,762,717	88.3	27,260	99.1
Neem leaf extract (200 µg/ml)	6,727,689	6,000,047	89.2	27,344	99.5
Neem leaf extract (300 µg/ml)	7,620,025	6,621,474	86.9	27,397	99.6

### Interaction network of genes and pathways picked from the gene expression and shRNA screening assays

We combined the short-listed genes whose expression valueswere significantly and serially up- or down-regulated (ratio at least below 0.8 or at least above 1.2) by the neem leaf extract over at least four consecutive time-points (Table S2) and those derived from the functional shRNA screening with significant changes in the clonal sizes of shRNA-infected cells in the neem leaf extract treatment, resulting in a consolidated list of 321 non-redundant genes (Table S5). These genes were considered to be affected somehow by the neem leaf extract treatment either or both in the functional and/or expression aspects. Thus, we further used this list for pathway and interaction network analyses to identify any molecular pathways influenced by the neem leaf extract.

**Figure 4:**
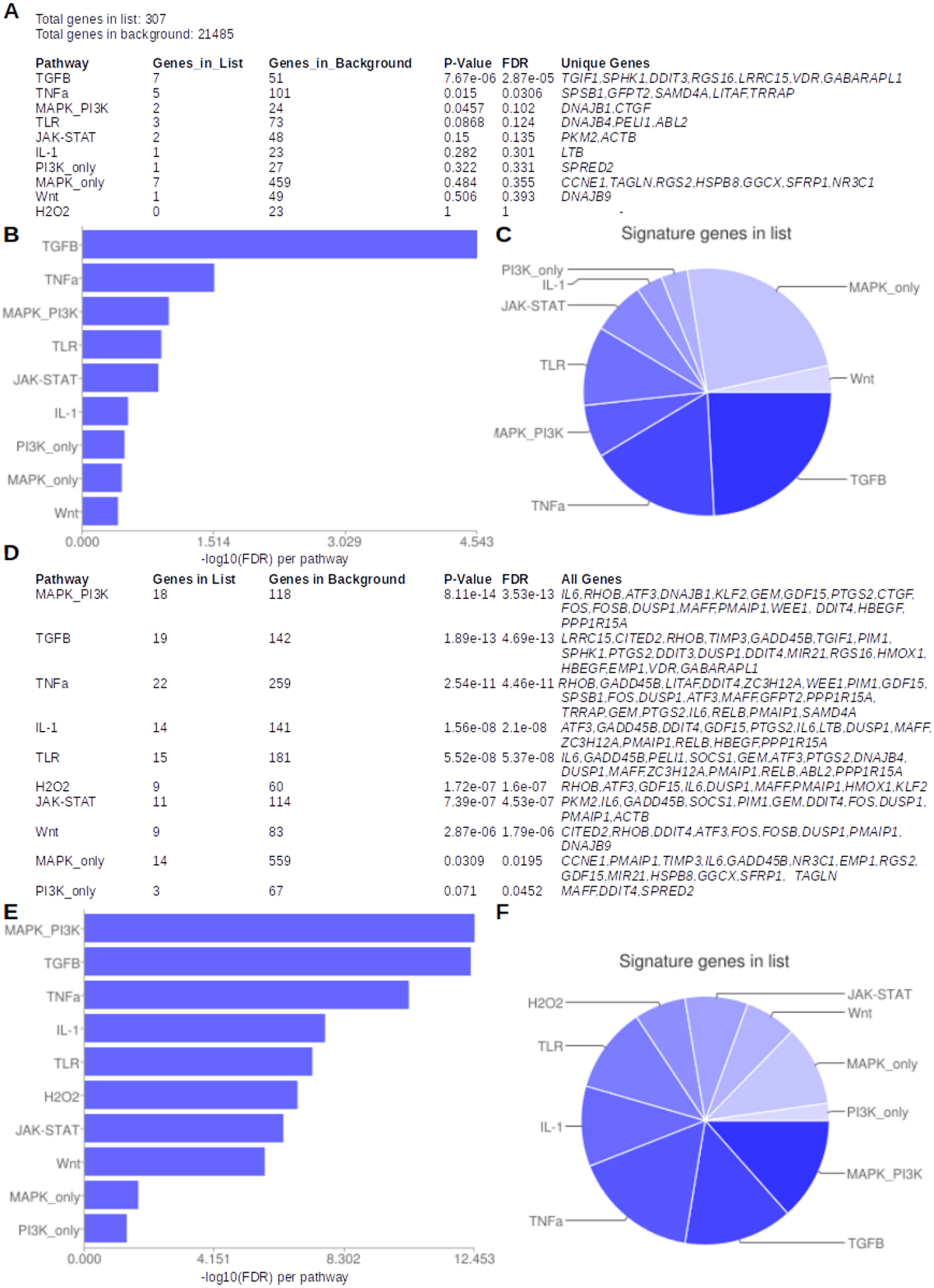
Enriched pathways and over-represented genes analysed by Speed using available options. (A–C) Using unique genes and (D–F) using all genes.

**Figure 5:**
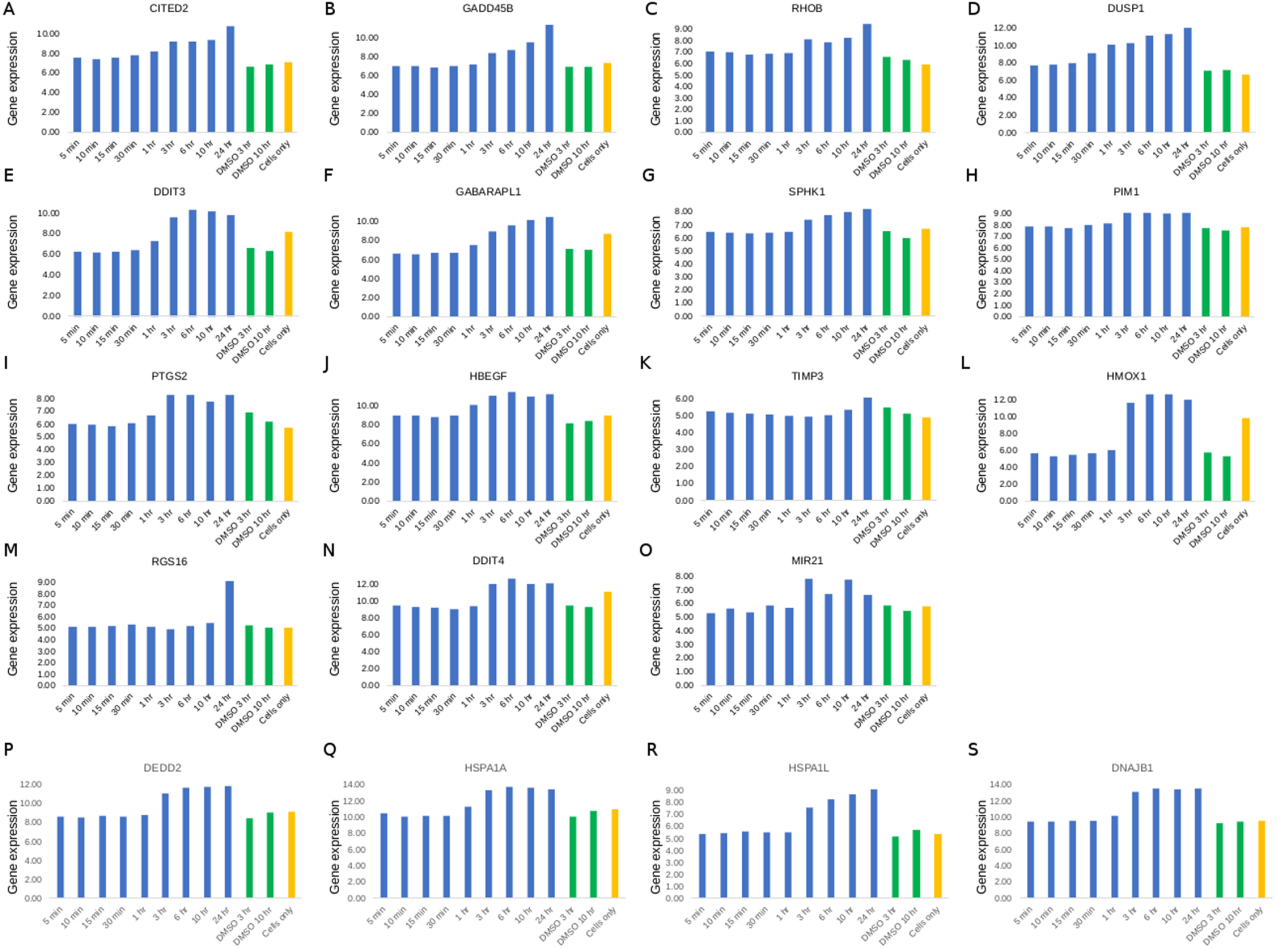
Whole genome microarray gene expression profiles. TGF-β (A–O) and HSF-1 (P–S)-related genes identified by the pathway analyses. Blue, green and orange bars represent crude neem leaf-treated, DMSO-treated and cell-only, respectively, as indicated on the *x*-axis.

The list of 321 genes (Table S5) was subjected to pathway enrichment analyses, using Speed and Reactome analysis tools, independently. The enrichment analyses using Speed while investigating only the unique set of signature genes resulted in only one pathway, the TGF-β signaling pathway*,* with a significant FDR adjusted *P* value at the 95% level of confidence (Fig. 4A). The unique genes perturbed in this pathway were *SPHK1*, *DDIT3*, *RGS16*, *LRRC15*, *VDR, TGIF1* and *GABARAPL1*. When all genes in the pathways were investigated, all signalling pathways passed the FDR significance threshold, with MAPK_PI3K signalling pathway at the top, with the least FDR and 18 genes perturbed out of 118 background genes, closely followed by TGF-β with 19 genes perturbed out of 142 background genes (Fig. 4B). The pathway enrichment analyses using Reactome resulted in three pathways with significant FDR (<0.05): Attentuation phase, *HSF-1*-dependent transactivation and *HSF-1* activation (Table S6). The genes over-represented in the “Attenuation Phase” pathway network were a subset of the two other *HSF-1*-related pathways; therefore, the *HSF-1*-related pathway was considered to be the enriched pathway in relation to the neem leaf extract treatment. The perturbed genes common to both *HSF-1*-related pathways were *DNAJB1*, *HSPA1L*, *HSPA1A* and *DEDD2. HSPB8* was unique to the *HSF-1*-dependent transactivation pathway network, while *RPA1* and *RPA2* were unique to the *HSF-1* activation pathway. *BAG3*, *PLK1* and *SPHK1* were the interactors used in the analysis in the case of *HSF-1* activation pathway (Table S6). Whole-genome microarray gene expression profiles in cells treated with the neem leaf extract showed activation of TGF-β (Fig. 5A) and HSF-1 (Fig. 5B)-related genes. Among the genes mapped to the TFG-β pathway, *DUSP1* was the only gene up-regulated post 30 min of treatment with neem leaf extract, another five genes (*CITED2*, *DDIT3*, *GABARAPL1*, *PTGS2* and *HBEGF*) were up-regulated post 1 h, seven genes (*GADD45B*, *RHOB*, *SPHK1*, *PIM1*, *HMOX1*, *DDIT4* and *MIR21*) up-regulated post 3 h, and two genes (*TIMP3* and *RGS16*) up-regulated only post 24 h (Fig. 5A). Out of these, the activation was most pronounced in magnitude for *HMOX1* gene (>2-fold), at the end of 3 h of treatment (Fig. 5A). In all four genes (*DEDD2*, *HSPA1A*, *HSPA1L* and *DNAJB1*) mapped to the HSF-1 pathway, a gradual or constant up-regulation effect was observed post 3 h of treatment (Fig. 5B).

### Identifying potential drug targets for neem leaf extract

**Figure 6:**
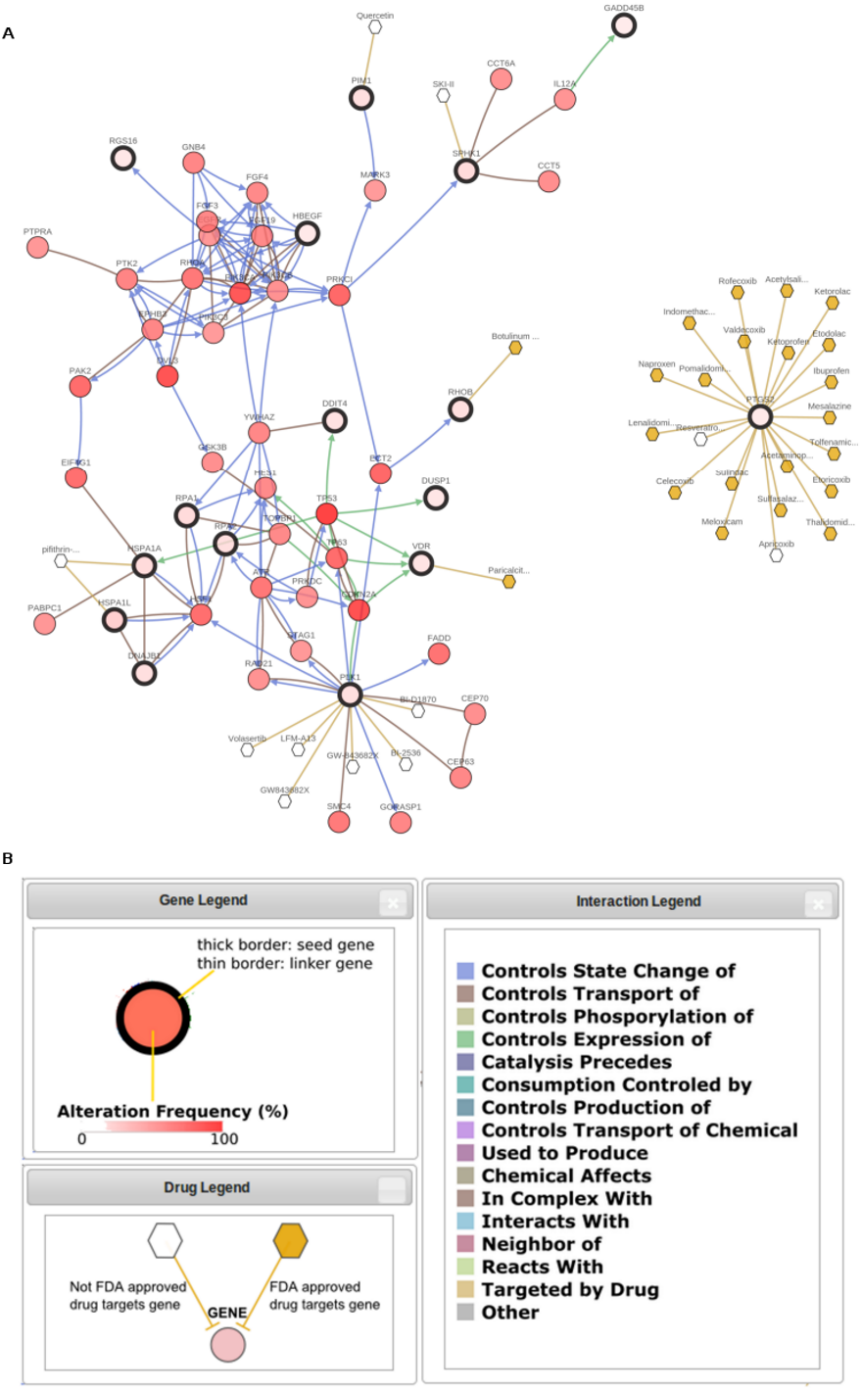
Hypothetical gene-drug interaction network in relation to the effect of neem extract in cancer. The (A) network and (B) legends plotted using cBioPortal (Gao et al., 2013; http://www.cbioportal.org) are represented here.

Finally, we explored any candidate molecular targets that may work in synergy with the neem extract for HNSCC patients. Depending on the list of genes in the pathways which were considered to be related to the mode of actions of neem extract (Fig. 4), we obtained the network and drug target interaction information for the 19 perturbed genes (*LRRC15*, *CITED2*, *RHOB*, *TIMP3*, *GADD45B*, *TGIF1*, *PIM1*, *SPHK1*, *PTGS2*, *DDIT3*, *DUSP1*, *DDIT4*, *MIR21*, *RGS16*, *HMOX1*, *HBEGF*, *EMP1*, *VDR* and *GABARAPL1*) from the TGF-β signalling pathway as detected by Speed (Parikh et al., 2010), the 10 genes (*DNAJB1*, *HSPA1L*, *HSPA1A*, *DEDD2*, *HSPB8*, *RPA1*, *RPA2*, *BAG3*, *PLK1* and *SPHK1*) from the *HSF-1*-based pathways as detected by Reactome, (Croft et al., 2013) and the 50 most altered genes for the TCGA HNSCC provisional data from the cBioPortal (Gao et al., 2013) (http://www.cbioportal.org). A network analysis was done using cBioPortal. The networks contained 55 nodes, including 16 out of 28 query genes (Fig. 6). Thirty-one percent of the network was categorized as ‘drug-target interactions’. In these networks, *PTGS2*, *VDR*, *RHOB*, *PIM1*, *HSPA1L*, *HSPA1A,* and *SPHK1* were used as targets for cancer drug development, previously. Out of these, *RHOB*, *VDR,* and *PTGS2* were the only candidates with FDA-approved anti-cancer drugs, with one each for *RHOB* and *VDR*, and 20 for *PTGS2* (Table S7). For others, quercetin (targeting *PIM1*) underwent seven clinical trials; and apricoxib (targeting *PTGS2*) underwent five clinical trials. Other genes such as *HMOX1* were the targets of eight non-anti-cancer non-FDA-approved drugs (Table S7).

## Discussion

Natural products are an important source of anti-cancer compounds (Hollosy & Keri, 2004). Neem leaf extract and derived metabolites are shown to act as anti-cancer compounds, specifically through the modulation of expression of critical cancer-related signalling molecules, including in oral cancer animal models (Harish Kumar et al., 2009; Paul, Prasad & Sah, 2011; Hsieh et al., 2015; Kasala, Bodduluru & Barua, 2015; Patel et al., 2016; Kowshik et al., 2017). Additionally, it is shown to act through modulation of the proinflammatory microenvironment in colorectal cancer (Gupta et al., 2013). Purified neem compounds have been shown to induce cytotoxicity and apoptosis, and affect cell cycle in various cancer cell lines (Roy et al., 2007; Priyadarsini et al., 2010; Chien et al., 2017), decrease tumor incidence and pre-neoplastic lesions in live hamster models of oral oncogenesis (Harish Kumar et al., 2010). This study aimed to identify the molecular function of the neem leaf extract with a particular focus on its anti-HNSCC property.

A critical application of the RNA interference-based functional screening is the identification of drug mechanisms as well as the synergic drug target(s). On the other hand, global time-course gene expression profiling under drug treatment also provides us significant insights into the drug mechanisms. In the current study, we combined these two independent global experiments to investigate the molecular mechanisms of the neem leaf extract with focuses on the anti-cancer properties for HNSCC. Studies combining shRNA and whole genome expression assays to identify potential drug targets have been shown in the past to yield useful results (Leung et al., 2016; Mendes-Pereira et al., 2012). The primary reason for using such a method in the current study was an anticipation that results from the shRNA screen would complement results from the gene expression studies, leading to more precise identification of pathways/targets.

Pathway enrichment analyses were conducted for shortlisted genes which were considered to be differentially affected somehow by the neem leaf extract, either or both in functional and/or gene expression aspects, identifying a possible role for the TGF-β signaling pathway and the *HSF-1* activation network. The *HSF-1* is related to the TGF-β signaling pathway and connects the IGF, TGF-β and cGMP pathways, and controls development processes (Barna et al., 2012). The aim of this study was to explore potential candidate genes/pathways that may work in synergy with the neem leaf extract. Precise molecular interactions and biochemical downstream effects of the neem in combination with those pathways, if any, are subject of our future research. While TGF-β and HSF-1 pathways have been already known as targetable in HNSCC by experimental means, our analyses provided a hint for the first time that the anti-cancer properties of crude neem leaf extract might function *via* these pathways. We further raised a list of potential anti-HNSCC drug targets among genes in the TGF-β signaling pathway and/or the *HSF-1* activation network (Table S7) that may work in synergy with the neem extract against HNSCC. Although the potential effects of these drugs in synergy with the neem leaf extract for HNSCC were not explored in this study, and are only a speculation at this point, it is highly warranted to confirm their potential applicability in the clinical settings for HNSCC patients.

Despite the power of the pooled shRNA library screening, there have been caveats to the shRNA-based drug target discovery, and our study is no exception. Regarding the individual shRNA behavior in the shRNA screening, one would realize some biological discrepancies in comparison with the global gene expression and integrated pathway analyses. For example, although our pathway analyses identified the possible synergic interaction between neem leaf extract and the TGF-β pathway, results from some of the individual hits in the shRNA screening were not concurrent. In the shRNA screening, knockdown of *SPHK1* and *LRRC15* (both are TGF-β pathway-related genes), for instance, were found to show no and positive effects on the cell growths of HSC-4 cells, respectively, under treatment of neem leaf extract (Table S4). It can be hypothesized that the biological effects of the shRNA-mediated inhibition of single gene are heavily influenced by numerous experimental conditions: such as the time-scale (24 h in gene expression and six days in shRNA screening) and chemical activity (long-term culture in shRNA screening may inactivate the extract). One must also consider that single gene inhibition within a global web of signaling networks may result in various levels of different biological homeostasis in the cells, resulting in cellular phenotypes different from those expected from the function of the pathway itself. Also, any gene has multiple down-stream effectors, possibly influencing both activation and inhibition of cell growth simultaneously. Despite such limitations in our shRNA screening, we identified potential candidate pathways by utilizing an integrated gene set obtained from either or both of the gene expression and/or shRNA screening. This signifies the importance of combining multiple global datasets and screening hits to identify candidate pathways of drug compounds with broad spectrums of biological effects such as the neem leaf extract. The current study attempts to broadly identify the known and novel gene targets of total crude neem leaf extract in relation to its anti-cancer effects on an HNSCC cell line. There are a few known purified chemical compounds derived from neem which function as anti-cancer molecules, such as the nimbolide. However, it is known that nimbolide alone cannot fully phenocopy the global anti-cancer properties of neem. Therefore, the crude neem leaf extract is anticipated to have a much wider range of anti-cancer properties. The current study is a proof-of-concept investigation using crude neem leaf extract to broadly uncover any unknown and/or novel signaling pathways and highlight a potential modality of its anti-cancer action. Although the current study identified the pathways involved, the effect may be a synergistic one between different compounds within the crude neem leaf extract. Future focused experiments with various purified neem compounds will shed further light on genes/pathways involved due to the action of any specific compounds.

## Conclusions

In summary, our study points out that neem leaf extract may offer a therapeutic potential to treat patients with HNSCC through its synergic modulations of pathways via *HSF-1* activation and TGF-β signaling.

##  Supplemental Information

10.7717/peerj.6464/supp-1 File S1 MIAME checklist for whole genome gene expression microarray dataset

10.7717/peerj.6464/supp-2 Figure S1 Supplementary Figures S1: IC50 calculation using the xCelligence Real-time Cell Analysis (RTCA) DP instrument with various neem extracts and nimbolide on HSC-4 cells.
S2: HSC-4 Cells after lentivirus infection (A) and the RFP signals (B) detected by fluorescent microscope.

10.7717/peerj.6464/supp-3 Table S1 Expression values for all genes at various time points and rescue experiment along with fold change statistics and cancer-specific pathways

10.7717/peerj.6464/supp-4 Table S2 Short listing based on serial up- and down-regulation across atleast four time points

10.7717/peerj.6464/supp-5 Supplemental Information 1 Tables S3 and S4 Table S3. Results from PCR amplification of the barcodes, in control and leaf-treated samples (200 ug/ml and 300 ug/ml), and ratios representing clonal size changes.
Table S4. Changes in clonal size from shRNA experiments with leaf extracts for drug target.

10.7717/peerj.6464/supp-6 Supplemental Information 2 Tables S5, S6 and S7 Table S5. Short listed genes from whole genome expression (WGGX) assay and shRNA experiments for pathway analyses.
Table S6. Results from pathway enrichment analyses performed using Reactome with a consensus list of 321 genes.
Table S7. Drug interactions.

## Additional Information and Declarations

### Data Availability

The following information was supplied regarding data availability: The microarray dataset from this study is available at NCBI GEO database: GSE125021.
