# Supplementary material for: Functional genomics screen with pooled shRNA library and gene expression profiling with extracts of Azadirachta indica identify potential pathways for therapeutic targets in head and neck squamous cell carcinoma
Source: PeerJ. 2019 Mar 1;7:e6464. doi: 10.7717/peerj.6464 (PMC6398373; doi:10.7717/peerj.6464)
Supplement: File S1 [file peerj-07-6464-s001.docx]

**Supplementary File S1. Miame Checklist**

**Krishnan et al.**

**Part 1 Experiment description**

- human Head and Neck Cancer cell line HSC-04

- experimental variables (time-dependent neem leaf treatment and rescue vs. DMSO treatment and rescue control)

- 5min, 10min, 15min, 30min, 1hr, 3hr, 6hr, 10hr, and 24hr

**Part 2 Array design.**

- Array series: GPL10904

- Array type: Illumina whole-genome HumanHT-12 v4 expression BeadChip

- Array size: 24 samples

- Number of probes: 47,231

**Part 3 Samples**

*- design:* RNA was extracted from HSC-4 cells, seeded and treated with neem leaf extract (200μg/ml) at nine pre-defined time points (5min, 10min, 15min, 30min, 1hr, 3hr, 6hr, 10hr, and 24hr) and rescued with fresh complete medium post 48-hour treatment at these time-points. The samples were assayed for gene expression using Illumina whole-genome HumanHT-12 v4 expression BeadChip (Illumina, San Diego, CA), following the manufacturer’s specifications.

*- growth protocol:* Cells were maintained in DMEM supplemented with 10% FBS, 1X MEM non-essential amino acids solution and 1X penicillin/streptomycin mixture (Gibco) at 370C with 5% CO2 incubation.

*- treatment protocol:* HSC-4 cells were seeded and treated with neem leaf extract (200μg/ml) at nine pre-defined time points (5min, 10min, 15min, 30min, 1hr, 3hr, 6hr, 10hr, and 24hr) and rescued with fresh complete medium post 48-hour treatment at these time-points. The samples were assayed for gene expression using Illumina whole-genome HumanHT-12 v4 expression BeadChip (Illumina, San Diego, CA), following the manufacturer’s specifications.

*- extract protocol:* RNA was extracted with Trizol reagent, followed by clean-up and DNase I treatment with QIAGEN RNeasy mini kit in accordance with the prescribed protocol provided with the kit. Quality control was performed with Agilent Bioanalyser.

*- label protocol:* The RNA samples were labeled using Illumina TotalPrep RNA Amplification kit (Ambion, USA) and processed following the manufacturer’s recommendations.

*- data processing:* Raw gene-wise expression signal intensities from GenomeStudio were transformed, normalized using VST (Variance Stabilizing Transformation) and LOESS methods, respectively, using the R package lumi (Du et al., 2008) and further batch-corrected using ComBat (Johnson et al., 2007).

**Part 4 Hybridizations**

*- hyb protocol:* Standard Illumina hybridization protocol

**Part 5 Measurements**

*- Which version of scanner software used: 1.5*

*- Laser power for scan: 532 nm and 660 nm dual-laser excitation*

*- Instrument model numbers: Illumina HiScan*

*- Normalization protocol: GenomeStudio*

*- Does the scanner software subtract background? How much? proprietary*

**Part 6 Normalization controls**

GenomeStudio was used.
