# Supplementary material for: Functional genomics screen with pooled shRNA library and gene expression profiling with extracts of Azadirachta indica identify potential pathways for therapeutic targets in head and neck squamous cell carcinoma
Source: PeerJ. 2019 Mar 1;7:e6464. doi: 10.7717/peerj.6464 (PMC6398373; doi:10.7717/peerj.6464)

**Supplementary Figure S1**. $IC_{50}$ calculation using the xCelligence Real-time Cell Analysis (RTCA) DP instrument with various neem extracts and nimbolide on HSC-4 cells.

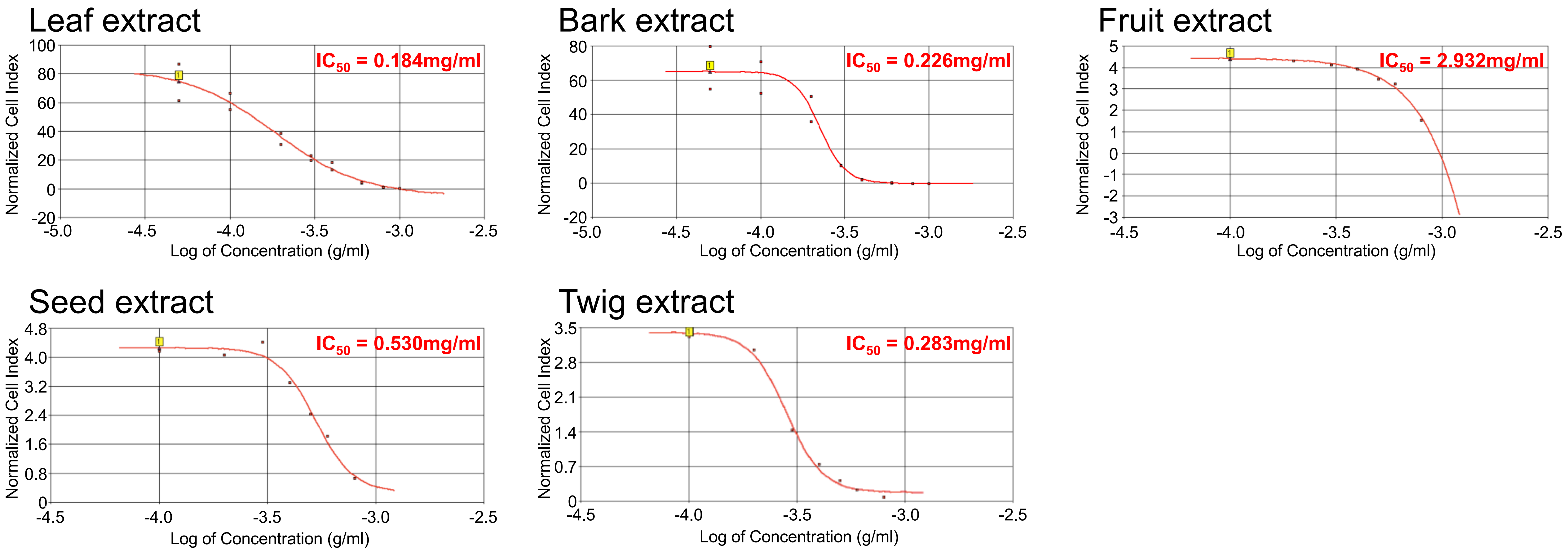

**Supplementary Figure S2**. HSC-4 Cells after lentivirus infection (A) and the RFP signals (B) detected by fluorescent microscope.

A B

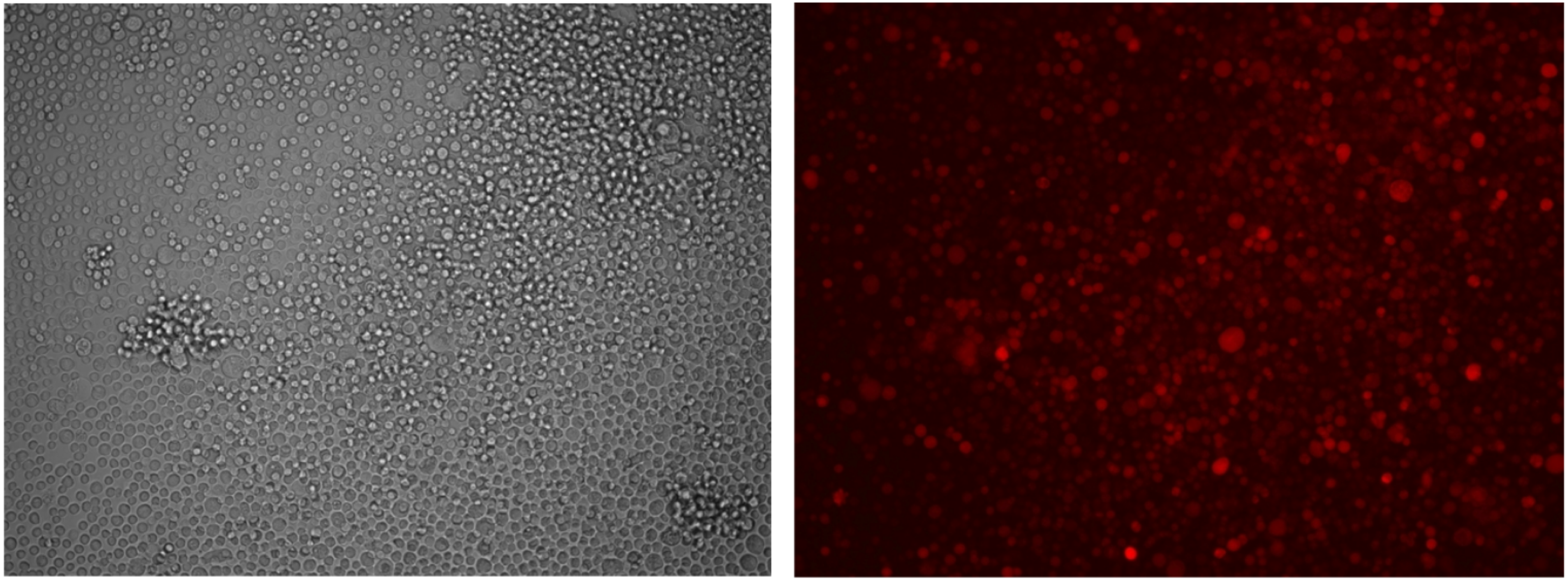

Supplement: Figure S1 — S1: IC50 calculation using the xCelligence Real-time Cell Analysis (RTCA) DP instrument with various neem extracts and nimbolide on HSC-4 cells. S2: HSC-4 Cells after lentivirus infection (A) and the RFP signals (B) detected by fluorescent microscope. [file peerj-07-6464-s002.pdf]
